# Kratom: a dangerous player in the opioid crisis

**DOI:** 10.1080/20009666.2018.1468693

**Published:** 2018-06-12

**Authors:** Khadija Tayabali, Colin Bolzon, Paul Foster, Janki Patel, Mohammad Omar Kalim

**Affiliations:** Internal Medicine, Greater Baltimore Medical Center, Towson, MD, USA

**Keywords:** Herb-induced liver disease, kratom, mitragynine, hepatotoxicity, opioid crisis, FDA, DEA, scheduled drugs

## Abstract

Kratom use as a herbal supplement is on the rise in the United States, with reported medical outcomes and lethal effects suggesting a public health threat. Even though the Drug Enforcement Administration has included kratom on its drugs of concern list and the FDA has published a press release to identify it as an opioid with a potential for abuse, its therapeutic and side effects are still not well defined in the literature. Here, we present a case of a 32-year-old man with a history of kratom use who became acutely ill with a brief prodromal illness, followed by jaundice and elevated liver enzymes showing a cholestatic picture, and his successful treatment. In this case, we emphasize the need for awareness of kratom exposure as a key contributor in the expansion of the opioid crisis, with therapeutic benefits earned at the expense of potentially lethal side effects.

## 1. Introduction

The current opioid epidemic is the deadliest drug crisis seen in American history and has left our nation grappling with the rising number of deaths fueled by opioid overdose [1], causing it to be declared a public health emergency [2].

Kratom (*Mitragyna speciosa*) is a tropical tree grown in Africa and Southeast Asia, which is currently gaining recognition in its contribution toward the opioid crisis. People from these regions have a long history of traditionally using the leaves of kratom to brew tea to help in managing pain and enhance productivity. In addition, the kratom plant has been noted to have dose-dependent stimulant and sedative effects, along with antinociceptive, antidepressant, anxiolytic, and anorectic effects [3].

Kratom misuse is an emerging trend in the Western world, and the wide availability of kratom on the Internet reflects extensive demand for this product. It is especially used by the online community to mitigate opioid withdrawal symptoms, self treat heroin/morphine dependence, and for pain relief in patients with chronic pain syndromes who feel stigmatized asking for help [4].

It is available through online vendors between $10 and $40 per ounce of plant material sold as a supplement in form of powder or capsules thus posing as an economical alternative to other expensive opioid-replacement medications, such as buprenorphine. Most importantly due to the loose regulation by the FDA, it has gained interest in the market due to its ability to be obtained without a prescription [4].

## 2. History

32-year-old Caucasian male with past medical history of hypertension, anxiety, and low back pain who presented to the Emergency Department (ED) with yellow looking skin associated with nausea, fatigue, joint pains, and night sweats of 2 weeks duration. He reported associated pale stools and dark urine, but denied any pruritus or changes in weight or appetite.

For his chronic low back pain he required occasional acetaminophen use, and more recently the use of kratom herbal supplements, both powder and tablet form. He had completed a dose of 60 tablets over 1 week (as per recommended dose on the bottle).

He had no known drug or environmental allergies. He had no smoking history, but drank alcohol occasionally. No illicit drug use. No sick contacts, recent hospitalizations, or any recent travel. He lived with his girlfriend and had been in a monogamous relationship for the last 5 years. His parents and siblings were healthy with no major medical concerns.

On admission to our facility, patient was clinically stable with a slightly elevated blood pressure of 141/82. Other vital signs were within normal limits. The patient was awake, alert, and fully oriented. Physical examination revealed jaundiced skin and icterus in the sclera and oral mucosa. Labs were significant for an elevated total bilirubin (6.3) and a cholestatic pattern of liver injury (as seen in Table 1), despite normal albumin and prothrombin time/International Normalized Ratio.

**Table 1.** Pertinent lab values.

	Reference range	Admission Day 1 Morning	Day 1 Late evening	Day 2	Day 3 Discharge
AST IU/L	4–37	222	198	167	136
ALT IU/L	4–40	365	334	359	299
ALP IU/L	36–117	391	362	372	314
Total bilirubin mg/dL	< 1	6.3	4.9	4.5	2.8
Direct bilirubin mg/dL	< 0.3		3.7	2.9	

Hepatitis Serology Panel nonreactive for hepatitis A, C, B surface antigen; positive for hep B surface antibody. HIV1/2 antibody nonreactive. CBC remained unchanged throughout hospital stay.

## 3. Differential diagnosis

Herb-induced liver injury (HILI) occurs in a small number of susceptible individuals. Assessment of causality should be performed using the Council for International Organizations of Medical Sciences Scale (CIOMS), which is specific for the liver and validated for hepatotoxicity, with points awarded for seven different components [5]. To make this diagnosis, it was necessary to rule out several intra- and extrahepatic causes.

Acetaminophen and salicylate levels were undetectable, and urine drug screen for common drugs of abuse with hepatotoxic potential (amphetamines, benzodiazepines, and cocaine) was negative.

Viral hepatitis markers, antibodies for autoimmune hepatitis, and rarer causes like ceruloplasmin for Wilson disease and alpha one antitrypsin levels were checked and were negative.

Abdominal ultrasound images revealed a normal sized liver with no focal abnormalities, decompressed gallbladder without gallstones, and non-dilated common bile and intrahepatic ducts ruling out biliary tract disease.

The patient scored 8 on the CIOMS scale (see Appendix for details), suggesting a high probability of his presentation being due to HILI and kratom use. Liquid mass chromatography spectrometry test – which is highly specific in detecting kratom metabolites in the urine – was positive (as noted in Table 2), further confirming our suspicion of kratom and its association to HILI.

**Table 2.** Kratom lab values.

	Reference range	Patient sample 12/08/17
Mitragynine	0 ng/mL	47.8 ng/mL
7-OHMG (Qualitative)	Negative	Positive

Performed at: 01 – MedTox Laboratories Inc, 402 W County Road D, St Paul, MN 551123522

## 4. Hospital course

Patient was thus admitted to the inpatient unit for a full work up of his acute liver injury. While labwork was pending, a literature search showed several case reports of patients presenting with acute hepatitis thought to be induced by kratom tea who benefited from N-acetylcysteine (NAC) [6]. Patient therefore received a loading dose of 150 mg/kg/h of NAC, but developed an anaphylactic reaction and subsequent doses were held.

During subsequent hospital days, liver enzymes started trending down, jaundice resolved, and the patient reported feeling better. Upon discharge, liver enzymes had not normalized, but he was considered stable and safe for discharge and outpatient follow-up.

## 5. Discussion

Epidemic levels of opioid abuse have been on the rise causing our nation to struggle with addiction, withdrawal, and opioid-related deaths. It is thus imperative that those grappling in the throes of addiction have access to FDA-approved medication and treatment options, rather than seeking out unregulated substances. Kratom is one such drug that has gained recent interest due to its opioid-like properties, which have led to its misuse to self-treat opioid dependence, for pain relief, and for its euphoric effects.

The active components in kratom, mitragynine (MG), and 7 hydroxymitragynine (7-OHMG) act as partial μ and Δ opioid receptor agonists, with the μ mediating euphoria, analgesia, and respiratory depression. This explains its analgesic effect and its role in ameliorating opioid withdrawal symptoms. The *M. speciosa* leaf extracts are its most effective part and have been shown to interact with the drug-metabolizing enzymes, showing the most potent inhibition on CYP2D6 activity [4,7].

The clinical manifestations of kratom are not well defined and studies are limited, but its safety profile has become a national and international concern, primarily due to excessive consumption being linked to increase in hospital visits for hepatic injury, seizures, coma, and death [3,8–10]. According to the Morbidity and Mortality weekly report by CDC, the number of reported exposure calls to poison centers related to kratom use increased 10-fold, from 26 in 2010 to 263 in 2015 [1]. The FDA recently put out a press release referring to kratom as an opioid and has so far noted up to 44 deaths related to its use [11].

The first reported case of kratom inducing liver damage was in a 25-year-old patient that developed fever, abdominal pain, and jaundice 10 days after the use of a kratom-based powder. Intake ranged from initial dose of 4.6–7 g/day to 8.6–14 g/day before onset of symptoms. Liver function tests showed a cholestatic picture, with intrahepatic cholestasis seen on biopsy. All other causes were ruled out. Upon discontinuation of the powder, he developed insomnia and restlessness, possibly as a sign of withdrawal [10]. Dorman et al. reported another case of a 58-year-old Caucasian man with prolonged kratom use who presented with jaundice and hepatotoxicity that resolved on withholding of the offending agent, with subsequent cholestatic liver injury resulting in readmission after he began using the same supplements again. All known causes of hepatotoxicity were ruled out [8].

Murine models fed doses as low as 100 mg/kg and as high as 1 g/kg of MG were noted to have multiorgan changes including elevation of transaminases with simultaneous lobular destruction, dilated biliary systems, and hemorrhagic hepatocytes demonstrated on biopsy [12,13].

## 6. Conclusion

Herbal products are overused as self medication due to the presumption that they are safe and without any compromising health effect.

Even though kratom has been illegal in Thailand since 1946 and criminalized in Australia in 2005, it is not currently scheduled in the United States. In August 2016, the DEA announced their intent to classify kratom as a Schedule I controlled substance eliciting a strong public outcry from those using kratom for chronic pain relief and as a control to their opioid addiction thus leading to withdrawal of this intent.

However, concern about the safety of this drug has continued to grow, and the FDA recently released a press statement about the ‘deadly risks’ associated with kratom (classified as an opioid) and citing its potential for abuse, addiction, and serious health consequences including death [11].

Given the lack of adequate data on the effects of the mitragynine species and our poor understanding of its potentially fatal adverse effects, further investigations are vitally needed.

## Appendix: CIOMS scale for acute drug reaction

**Table:**

Cholestatic pattern of liver injury			
	First Exposure	Second Exposure	Points
Time to onset of injury: a. from the beginning of the drug b. from cessation of the drug	**5-90 days**	1-15 days	**2**
	<5 or >90 days	>15 days	1
	**≤ 30 days**	**≤ 30 days**	**1**
Risk factors	Alcohol		1
	Age > 55		1
Course of the reaction	**≥ 50% improvement within 180 days**		**2**
	< 50% improvement within 180 days		1
	Persistence or increase or lack of information		0
Concomitant therapy	**None**		**0**
	Suggestive to onset of injury		-1
	Known hepatotoxin with suggestive time to onset of injury		-2
	Clear evidence for its role		-3
Other Causes	**Ruled out**		**2**
	Probable		-3
Previous Hepatotoxicity Info	Published and labelled		2
	**Published not labelled**		**1**
	Unknown		0
Response to reexposure	**Not done**		**0**

Likelihoods:  >8 Highly probable,  6-8 Probable, 3-5 Possible, 1-2 unlikely, 0 excluded
